# Geographical, Ethnic and Socio-Economic Differences in Utilization of Obstetric Care in the Netherlands

**DOI:** 10.1371/journal.pone.0156621

**Published:** 2016-06-23

**Authors:** Anke G. Posthumus, Gerard J. Borsboom, Jashvant Poeran, Eric A. P. Steegers, Gouke J. Bonsel

**Affiliations:** 1 Department of Obstetrics and Gynaecology, Division of Obstetrics and Prenatal Medicine, Erasmus University Medical Centre, Rotterdam, the Netherlands; 2 Department of Public Health, Erasmus University Medical Centre, Rotterdam, The Netherlands; 3 Department of Healthcare Policy and Research, Division of Biostatistics and Epidemiology, Weill Cornell Medical College, New York, United States of America; St Francis Hospital, UNITED STATES

## Abstract

**Background:**

All women in the Netherlands should have equal access to obstetric care. However, utilization of care is shaped by *demand* and *supply* factors. Demand is increased in high risk groups (non-Western women, low socio-economic status (SES)), and supply is influenced by availability of hospital facilities (hospital density). To explore the dynamics of obstetric care utilization we investigated the joint association of hospital density and individual characteristics with prototype obstetric interventions.

**Methods:**

A logistic multi-level model was fitted on retrospective data from the Netherlands Perinatal Registry (years 2000–2008, 1.532.441 singleton pregnancies). In this analysis, the first level comprised individual maternal characteristics, the second of neighbourhood SES and hospital density. The four outcome variables were: referral during pregnancy, elective caesarean section (term and post-term breech pregnancies), induction of labour (term and post-term pregnancies), and birth setting in assumed low-risk pregnancies.

**Results:**

Higher hospital density is not associated with more obstetric interventions. Adjusted for maternal characteristics and hospital density, living in low SES neighbourhoods, and non-Western ethnicity were generally associated with a lower probability of interventions. For example, non-Western women had considerably lower odds for induction of labour in all geographical areas, with strongest effects in the more rural areas (non-Western women: OR 0.78, 95% CI 0.77–0.80, p<0.001).

**Conclusion:**

Our results suggest inequalities in obstetric care utilization in the Netherlands, and more specifically a relative underservice to the deprived, independent of level of supply.

## Introduction

It is generally accepted that all individuals should have equal *access* to health, and in order to attain this, equal *access to health care* [1, 2]. Equity in access to health care means equal access to care for people with equal conditions (*horizontal* equity) [3]. In this context all pregnant women in the Netherlands have universal obstetric care access, regardless of insurance status or legal status (e.g. asylum seekers). Furthermore, women with higher risks for adverse outcomes qualify for obstetric care in hospitals instead of at community midwifery practices. This is known as *vertical* equity, in which the presence of severer conditions justifies the availability of more resources [3].

Even with *horizontal* and *vertical* equity theoretically in place, access to and utilization of health care is also determined by the interaction between *demand* and *supply* factors [4]. *Demand* factors refer to health risks and health behaviours, including individual factors associated with higher risk for disease and more utilization of care. In the context of obstetric care, being of non-Western ethnic descent and living in deprived neighbourhoods are acknowledged demographic demand factors [5]. This is also true for the Netherlands [6, 7]. Supply factors in brief include the availability of care and its perceived quality[4]. One important supply factor is the geographical density of health care facilities. Density of these facilities often differs between urban and more rural areas, where living in a rural area often results in longer travelling distances and thus comprised access. Conversely, a high hospital density in the absence of other barriers bears with it the risk of causing *supplier induced demand*, with resulting unnecessary medical interventions [8, 9].

This study investigated the utilization of obstetric care in the Netherlands, hypothesizing that higher hospital density is associated with an increased number of obstetric interventions (increased *supply*). A second hypothesis was that -due to individual risk patterns- 1) the probability of obstetric interventions is higher in non-Western women due to their increased risk of adverse outcomes compared to Western women, and 2) that at a higher aggregation level this similarly applies to women living in deprived neighbourhoods compared to women outside of these neighbourhoods (increased *demand*).

To test these hypotheses, we conducted multilevel analyses on the association of hospital density (*supply*) and individual level determinants (*demand*) with four prototype obstetric care interventions in the Netherlands. The analyses were performed separately for large urban areas, medium-sized urban areas and more rural areas.

## Methods

### General

**Fig 1:**
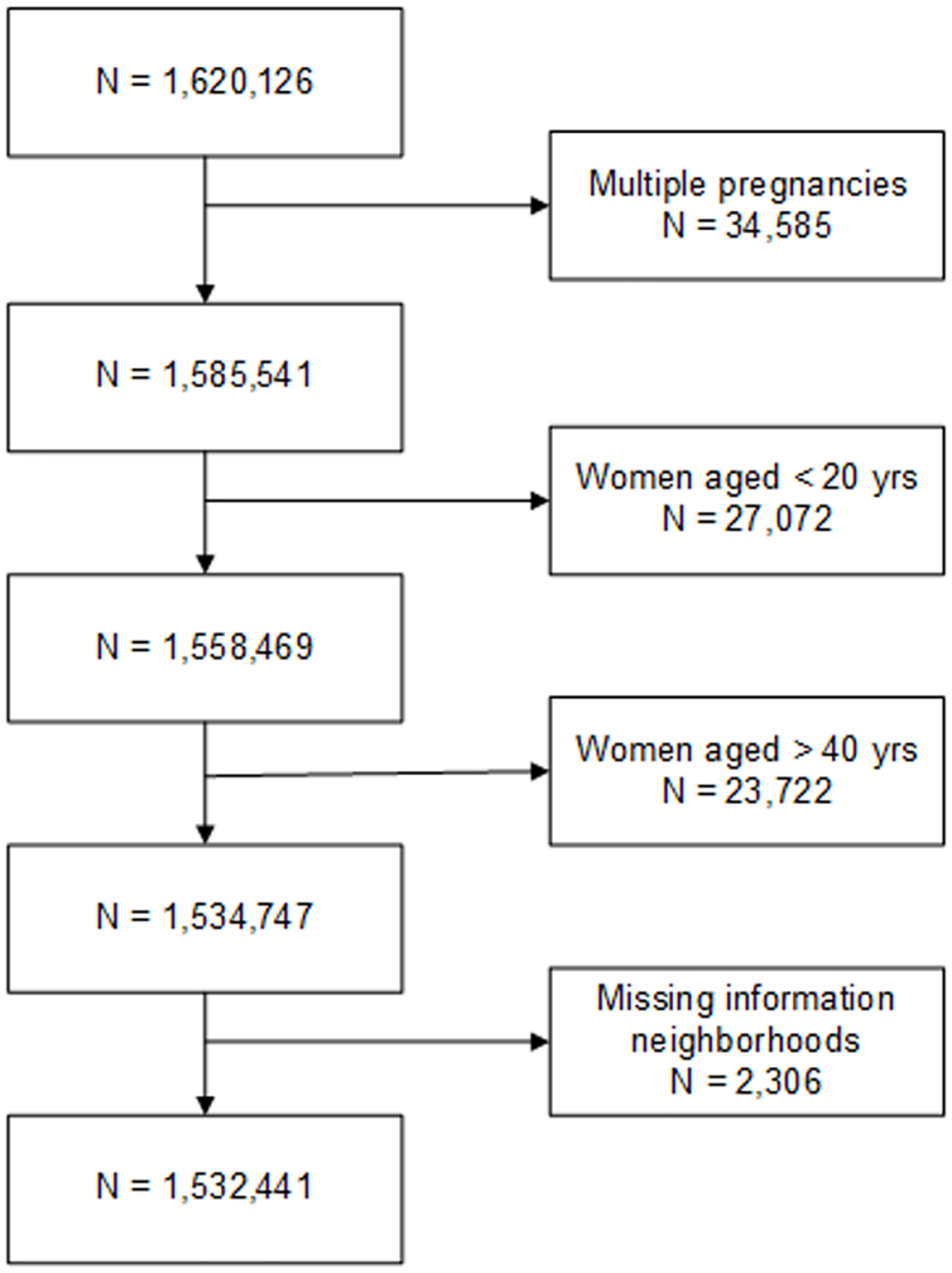
Flowchart of women excluded from the study.

We conducted a multi-level observational study using retrospective data of all singleton births in the years 2000 to 2008 (n = 1.532.441) in the Netherlands to investigate factors affecting utilization of obstetric care (see Fig 1 for exclusions). The national dataset on which we conducted secondary analyses was made available by the Netherlands Perinatal Registry (which covers over 97% of all pregnancies)[10]. The use of the anonymized patient data for this study was approved by the Netherlands Perinatal Registry (project number 12.67) (additional information on the registry: www.perinatreg.nl/home_english). Written consent from pregnant women was not needed as the registry protects their anonymity. This research received no specific grant from any funding agency in the public, commercial, or not-for-profit sectors. We linked data on neighbourhood hospital density and neighbourhood socio-economic status to individual perinatal records using the 4-digit postal codes.

Two levels of aggregation were defined. The first level consisted of individual maternal characteristics, and the second level of neighbourhood SES and neighbourhood hospital density. The four interventions used to represent utilization were selected because they cover different stages of pregnancy. The independent and dependent variables are described in more detail below.

### Independent variables

#### First level: Individual Characteristics

Maternal age, parity and ethnicity were included. Maternal age was categorized into 20 to ≤ 24 years, 25 to ≤ 29 years, 30 to ≤34 years and ≥35 years. We categorized parity (prior births) into 0, 1, 2–3 and ≥4 births respectively. Maternal ethnicity was recorded by the obstetric care provider and was based on either self-declared ethnicity, race, or country of birth of the mother or her parents. It is not possible to retrace on which of these criteria the caregiver has based the filled out ethnicity for each individual woman. Because of the implied heterogeneity of definitions, we have dichotomized ethnicity into ‘Western’ or ‘non-Western’ ethnicity.

We excluded multiple pregnancies (n = 34,585; 2.1%), women under 20 (n = 27,072; 1.7%) or over 40 years of age (n = 23,722; 1.5%). These groups were excluded because of their a priori increased risks of adverse pregnancy outcomes [11–13]. Additionally, we excluded pregnancies with missing information on neighbourhood characteristics (n = 2306; 0.1%).

#### Second level: Neighbourhood Characteristics

Neighbourhoods were defined on the basis of the 4-digit postal code areas with on average 4000 inhabitants (±40 births annually), which are comparable in size to United Kingdom lower layer super output areas or United States of America Census tracts [14, 15]. Postal codes are commonly known as ZIP code in the USA. In our analyses we included two neighbourhood level determinants, neighbourhood socio-economic status (SES) and Neighbourhood hospital density.

Data on neighbourhood SES were obtained from the Netherlands Institute of Social Research [16]. A numeric SES-score created with principal component analysis is available for all 4-digit postal code areas with more than 100 inhabitants. The SES-score is updated every four years and for this study the scores for the year 2006 were used. The SES-score is based on the mean income per household, % households with a low income, % unemployed inhabitants and % households with an on average low education. The variance explained by the first principle component is 51.1%. There is a strong negative association between SES status and the % of households with a low income. The same is the case for SES status and the % of unemployed inhabitants and the % of households with an on average low education. There is a strong positive association between SES status and the mean income per household. [17]. For the purpose of this study we categorized the continuous SES-scale into quintiles.

Neighbourhood hospital density (supply) was defined as the availability of hospital care specified per geographical area. It was calculated as the summation of all available hospital capacity (expressed as the average delivery volume per hospital per year). Each individual hospital’s capacity was discounted by the distance of each 4-digit zip code area to this hospital (‘zip code centroid approximation’). This discount factor was inversely quadratic: triple the distance implied 1/9^th^ of the capacity impact.

### Dependent variables

Four care interventions were selected as a proxy for obstetric care interventions in general. The cohort size differed for each analysis because each focuses on a different subpopulation. These subpopulations are also specified below.

#### 1. Referral during pregnancy from community midwife to obstetrician

Usually, midwives take care of women with an uncomplicated pregnancy, child birth and childbed and refer to an obstetrician if complications (threaten to) occur. This outcome indicator is defined as a dichotomous variable: referral versus no referral at any time during pregnancy before the start of labour.

#### 2. Induction of labour in non-breech term and post-term pregnancies (≥ 37 weeks of gestation)

Labour may be induced if pregnancies are post-term or because of predefined high risk medical conditions. This outcome indicator is defined as a dichotomous variable: induction versus no induction of labour.

#### 3. Elective caesarean section (CS) in term and post-term breech pregnancies (≥ 37 weeks of gestation)

In the Netherlands both a vaginal trial of labour (TOL) or an elective primary CS are accepted delivery options for a child in breech position. This outcome indicator is defined as a dichotomous variable: vaginal TOL or elective primary CS. Women who give birth by secondary CS (after the TOL failed) are assigned as vaginal TOL.

We deliberately used the CS rate in breech deliveries and not the overall CS rate, because the non-breech elective CS group and the emergency CS group, are both quite heterogeneous; sizeable true indication prevalence differences between hospital areas may exist, which are not covered by variables in the registry (e.g. there is no acute fetal risk information in the registry or fetal cardiotography outcomes) but additional policy differences may also be present. Due to the mixed background of CS rate differences in the non-breech elective CS group and the emergency CS group (both medical/clinical background and policy background), the interpretation of any outcome would be vulnerable for opportunistic criticism.

#### 4. Birth setting in low-risk pregnancies

Assumed low-risk pregnant women, can either deliver at home, in a birthing centre or in an out-patient clinic (located in a hospital) under supervision of their midwife. This outcome indicator is defined as a dichotomous variable: birth in an out-patient clinic (located in a hospital) or elsewhere (at home or in a birthing centre).

### Analytical Strategy

We employed multivariable multilevel logistic regression models with a random intercept for postal code areas. The GLIMMIX procedure in SAS version 9.3 was used for the analysis of the data of women (first level), nested within neighbourhoods (second level). GLIMMIX is a procedure for fitting generalized linear mixed models. These models allow for data that are not necessarily normally distributed. The model results are reported as odds ratios. First, we fitted a null-model to determine neighbourhood level variance, for all our outcome measures separately. To determine whether clustering was present we calculated the Intraclass correlation coefficient (ICC). If the ICC deviates from zero, the use of a multilevel model is appropriate [18]. We then fitted the full model for each care intervention, including an interaction term for parity*age.

The analyses were further stratified according to geographical area, because of known heterogeneity in population density and its interactions with the determinants included in the model. We distinguished three different types of areas: 1) urban areas, 2) semi-urban areas and 3) more rural areas. The first category contained all postal code areas in the four largest cities of the country (C4), the second contained the fifth up to and including the tenth largest city (C6) and the last contained all other areas (Cx). We have made the distinction between these groups of cities because in the Netherlands these distinctions are most often used in the political, scientific and policy fields. The cut-off at the C4 level was chosen because these large cities have significantly higher levels of adverse health outcomes, including perinatal outcomes in comparison to the rest of the country [19]. The number of postal code areas included in all analysis was 3422 (median 221, 20^th^ percentile -80^th^ percentile: 37–700).

## Results

A total of 1.532.441 singleton pregnancies were included in this study. Table 1 shows baseline characteristics of the study populations for the three geographical areas. Fifteen percent of births occurred in the four largest cities (C4). The highest numbers of non-Western women were recorded in the C4 (42%), as compared to 21% and 10% in C6 and Cx, respectively. At neighbourhood level, 54% of the women in the C4 were living in the lowest SES quintile neighbourhoods compared to 27% and 13% in the other areas. As expected, neighbourhood hospital density was highest in the C4 (1.05) and lowest in the Cx (0.61).

**Table 1:** Descriptive statistics of individual variables and obstetric care interventions.

	Total	Urban areas (C4)	Semi-urban areas (C6)	Rural areas (Cx)	P-value
**Average population density (people/km2)**	489	4,120	2,053		
	N (%)	N (%)	N (%)	N (%)	
Total number of cases	1,532,441	231,886 (15)	104,358 (7)	1,196,197 (78)	
**Individual determinants**					
*Maternal age*					<0.01
20 ≤ 24 yrs	161,800 (11)	34,705 (15)	12,285 (12)	114,810 (10)	
25 ≤29 yrs	456,108 (30)	61,134 (26)	30,294 (29)	364,680 (31)	
30 ≤34 yrs	620,399 (41)	85,097 (37)	41,652 (40)	493,650 (41)	
>34 yrs	294,134 (19)	50,950 (22)	20,127 (19)	223,057 (19)	
*Parity*					<0.01
Primiparous (no prior birth)	699,385 (46)	113,493 (49)	50,435 (48)	535,457 (45)	
Multiparous (1 prior birth)	558,658 (37)	74,538 (32)	37,393 (36)	446,727 (37)	
Multiparous (2–3 prior births)	247,818 (16)	37,978 (16)	15,034 (14)	194,806 (16)	
Multiparous (≥4 prior births)	26,580 (2)	5,877 (3)	1,496 (1)	19,207 (2)	
*Ethnicity*					<0.01
Western ethnicity	1,291,067 (84)	133,713 (58)	82,573 (79)	1,074,781 (90)	
Non-Western ethnicity	241,374 (16)	98,173 (42)	21,785 (21)	121,416 (10)	
**Neighbourhood determinants**					
*Socio-economic Status*					<0.01
0-20th percentile	305,922 (20)	126,222 (54)	28,565 (27)	151,135 (13)	
>20-40th percentile	306,888 (20)	28,424 (12)	25,948 (25)	252,516 (21)	
>40-60th percentile	306,133 (20)	17,276 (8)	8,212 (8)	280,645 (24)	
>60-80th percentile	308,175 (20)	18,817 (8)	12,933 (12)	276,425 (23)	
>80th percentile	305,323 (20)	41,147 (18)	28,700 (28)	235,476 (20)	
Hospital density (mean)	0.68	1.05	0.7	0.61	
**Obstetric care interventions**					
*Referral (during pregnancy)*					<0.01
Referral	522,946 (41)	83,089 (43)	39,957 (47)	399,900 (40)	
No referral	747,210 (59)	112,065 (57)	45,106 (53)	590,039 (60)	
*Location of low- risk births*					<0.01
Out-patient clinic	167,404 (34)	36,215 (52)	9,816 (35)	121,373 (31)	
Home	327,338 (66)	33,831 (48)	18,297 (65)	275,210 (69)	
*Primary Caesarean Section (breech)*					<0.01
Primary Caesarean Section	32,367 (54)	3,873 (52)	2,188 (55)	26,306 (54)	
Vaginal Trial of labour	27,400 (46)	3,545 (48)	1,786 (45)	22,069 (46)	
*Induction of labour (non-breech)*					<0.01
Induction	191,704 (14)	26,069 (13)	12,599 (14)	153,036 (14)	
No induction	1,171,639 (86)	180,617 (87)	80,191 (86)	910,831 (86)	

(Source: Netherlands Perinatal Registration, 2000–2008).

In the C4 48% of women delivered at home, as compared to 65% and 69% in the C6 and Cx, respectively. In term breech pregnancies a vaginal trial of labour (TOL) took place most often in the C4 (48%), followed by the Cx (46%) and C6 (45%). Concerning induction of labour, the differences according to geographical location were small.

### Multilevel logistic regression models

The ICC in the null-models deviated significantly from zero, justifying the use of multilevel analysis.

Table 2 shows the association of individual and neighbourhood level characteristics with referral during pregnancy from the community midwife to an obstetrician, for the total population and stratified according to geographical area. On the individual level, higher age and non-Western ethnicity were associated with higher odds of referral. Non-Western women were referred more often, irrespective of area of geographical location. While in the C4 the lowest SES group (least affluent) was referred less often, the reverse was true in the rural and semi-urban areas with lower SES groups being referred more often. Interestingly, nulliparous women in the C4 and C6 were referred more often than multiparous women (≥3 births), whilst the opposite was the case in the Cx.

**Table 2:** Multilevel logistic regression models of individual level maternal characteristics, neighbourhood SES and hospital density and referral during pregnancy°. (Odds ratios, 95% confidence intervals in parentheses).

N = 1,076,494 births	Total	C4^a^	C6^b^	Cx^c^
Individual level	OR (95%CI)	OR (95%CI)	OR (95%CI)	OR (95%CI)
Maternal age (Ref. = 30–34 yr)				
Overall	***	***	***	***
≤ 24yr	0.96 (0.88–1.04)	0.89 (0.76–1.05)	0.97 (0.75–1.26)	0.96 (0.86–1.07)
25-29yr	0.91 (0.89–0.94)	0.89 (0.84–0.95)	0.98 (0.88–1.09)	0.91 (0.88–0.94)
>34yr	1.32 (1.29–1.34)	1.31 (1.26–1.36)	1.38 (1.29–1.49)	1.31 (1.29–1.34)
Parity (Ref. = multiparous 1 prior birth)				
Overall	***	***	***	***
Nulliparous (0 births)	1.36 (1.34–1.37)	1.35 (1.32–1.38)	1.29 (1.24–1.33)	1.37 (1.35–1.38)
Multiparous (2–3 births)	1.05 (1.03–1.07)	1.16 (1.12–1.20)	1.19 (1.12–1.26)	1.01 (0.99–1.03)
Multiparous (≥3 births)	1.28 (1.17–1.40)	1.62 (1.37–1.92)	1.42 (1.08–1.86)	1.16 (1.04–1.31)
Ethnicity (Ref. = Western)				
Non-Western	1.11 (1.10–1.13)***	1.13 (1.11–1.16)***	1.19 (1.15–1.23)***	1.09 (1.07–1.10)***
Neighbourhood level				
Socio-economic status (Ref.> 80th percentile)				
Overall	***		*	***
0-20th percentile	1.12 (1.08–1.17)	0.92 (0.85–0.99)	1.14 (1.03–1.26)	1.15 (1.10–1.20)
>20-40th percentile	1.03 (1.00–1.07)	0.93 (0.84–1.03)	1.05 (0.95–1.16)	1.05 (1.01–1.09)
>40-60th percentile	1.00 (0.97–1.04)	0.90 (0.80–1.02)	1.04 (0.90–1.21)	1.03 (0.99–1.08)
>60-80th percentile	0.96 (0.93–1.00)	0.94 (0.84–1.05)	0.97 (0.85–1.10)	0.99 (0.95–1.03)
Density	1.08 (1.03–1.28)***	0.85 (0.75–0.96)***	0.94 (0.77–1.14)	1.07 (1.00–1.13)*

°Referral during pregnancy versus no referral, reference category.

Stratification for three geographical areas:

^**a**^C4 = Urban areas;

^**b**^C6 = Semi-urban areas;

^**c**^Cx = Rural areas. Levels of significance:

* = p<0.10;

** = p<0.05;

*** = p<0.01.

At the neighbourhood level, hospital density was associated with referral in the C4 and Cx only, demonstrating a negative association for the first and a positive association for the latter.

A similar analysis in Table 3 shows demand effects in the induction of labour in non-breech term pregnancies. Non-Western ethnicity was associated with considerably lower odds for induction in all geographical areas, with strongest effects in the Cx. The SES pattern however, resembled that of referral: higher odds of induction with lower SES in the C4 and Cx. Here, increased hospital density was associated with lower chances of induction in both the C4 and Cx.

**Table 3:** Multilevel logistic regression models of individual level maternal characteristics, neighbourhood SES and hospital density and induction of labour°. (Odds ratios, 95% confidence intervals in parentheses).

N = 1,363,343 births	Total	C4^a^	C6^b^	Cx^c^
Individual level	OR (95%CI)	OR (95%CI)	OR (95%CI)	OR (95%CI)
Maternal age (Ref. = 30–34 yr)				
Overall	***	***	*	***
≤ 24yr	0.88 (0.78–0.99)	0.85 (0.69–1.04)	0.69 (0.41–1.18)	0.90 (0.77–1.05)
25-29yr	0.99 (0.96–1.02)	0.90 (0.84–0.97)	1.00 (0.88–1.13)	1.01 (0.98–1.05)
>34yr	1.08 (1.06–1.11)	1.13 (1.08–1.18)	1.08 (1.00–1.18)	1.08 (1.05–1.10)
Parity (Ref. = multiparous 1 prior birth)				
Overall	***	***	***	***
Nulliparous (0 births)	1.21 (1.20–1.23)	1.33 (1.28–1.37)	1.25 (1.19–1.31)	1.19 (1.17–1.21)
Multiparous (2–3 births)	1.28 (1.25–1.31)	1.28 (1.22–1.35)	1.39 (1.28–1.51)	1.28 (1.25–1.31)
Multiparous (≥3 births)	1.59 (1.40–1.79)	1.74 (1.41–2.15)	1.28 (0.74–2.19)	1.57 (1.34–1.85)
Ethnicity (Ref. = Western)				
Non-Western	0.82 (0.81–0.83)***	0.93 (0.90–0.96)***	0.82 (0.78–0.87)***	0.78 (0.77–0.80)***
Neighbourhood level				
Socio-economic status (Ref.> 80th percentile)				
Overall	***	***		***
0-20th percentile	1.13 (1.08–1.18)	1.29 (1.16–1.44)	0.92 (0.82–1.03)	1.13 (1.07–1.19)
>20-40th percentile	1.13 (1.08–1.17)	1.22 (1.06–1.40)	0.92 (0.82–1.03)	1.12 (1.07–1.18)
>40-60th percentile	1.11 (1.06–1.16)	1.26 (1.07–1.48)	0.92 (0.78–1.09)	1.10 (1.05–1.15)
>60-80th percentile	1.03 (0.99–1.08)	1.17 (1.01–1.35)	0.96 (0.84–1.11)	1.02 (0.98–1.07)
Density	0.78 (0.74–0.83)***	0.79 (0.67–0.94)***	0.96 (0.77–1.20)	0.80 (0.75–0.86)***

°Induction of labour versus no induction of labour, reference category.

Stratification for three geographical areas:

^**a**^C4 = Urban areas;

^**b**^C6 = Semi-urban areas;

^**c**^Cx = Rural areas. Levels of significance:

* = p<0.10;

** = p<0.05;

*** = p<0.01.

Likewise Table 4 shows that non-Western women had substantially lower odds than Western women to receive a caesarean section (CS) in term breech pregnancies, particularly in the C4 (OR 0.86, CI 0.77–0.97). At neighbourhood level, effects were variable. A 25% decreased odds for a CS was observed in women from the lowest SES quintile in the C4. Higher levels of hospital density were associated with lower odds for a CS in the Cx (OR 0.86, CI 0.76–0.98) and approximately the same but weaker associations were present in the C4.

**Table 4:** Multilevel logistic regression models of individual level maternal characteristics, neighbourhood SES and hospital density and primary caesarean sections (CS)° in term breech pregnancies (≥37 weeks of gestation). (Odds ratios, 95% confidence intervals in parentheses).

N = 1,363,343 births	Total	C4^a^	C6^b^	Cx^c^
Individual level	OR (95%CI)	OR (95%CI)	OR (95%CI)	OR (95%CI)
Maternal age (Ref. = 30–34 yr)				
Overall	***	***	***	***
≤ 24yr	0.81 (0.69–0.96)	0.93 (0.67–1.30)	0.52 (0.25–1.09)	0.80 (0.65–0.97)
25-29yr	0.91 (0.86–0.97)	0.82 (0.69–0.98)	0.68 (0.51–0.91)	0.94 (0.88–1.01)
>34yr	1.22 (1.16–1.28)	1.20 (1.04–1.38)	1.11 (0.89–1.37)	1.22 (1.15–1.29)
Parity (Ref. = multiparous 1 prior birth)				
Overall	***	*	***	***
Nulliparous (0 births)	1.11 (1.06–1.18)	1.10 (0.96–1.25)	1.02 (0.83–1.25)	1.13 (1.06–1.20)
Multiparous (≥2 births)	0.67 (0.59–0.76)	0.84 (0.64–1.11)	0.38 (0.21–0.69)	0.66 (0.56–0.77)
Ethnicity (Ref. = Western)				
Non-Western	0.93 (0.88–0.99)**	0.86 (0.77–0.97)**	0.86 (0.70–1.05)	0.98 (0.90–1.06)
Neighbourhood level				
Socio-economic status (Ref.> 80th percentile)				
Overall	*	***		
0-20th percentile	0.92 (0.85–1.00)	0.75 (0.64–0.88)	0.85 (0.69–1.05)	0.99 (0.90–1.09)
>20-40th percentile	0.93 (0.87–1.00)	0.82 (0.68–1.00)	0.86 (0.70–1.06)	0.95 (0.88–1.03)
>40-60th percentile	0.95 (0.89–1.02)	1.00(0.80–1.26)	0.92 (0.68–1.24)	0.97 (0.89–1.05)
>60-80th percentile	0.90 (0.84–0.97)	1.04 (0.83–1.29)	0.86 (0.67–1.10)	0.91 (0.84–0.98)
Density	0.86 (0.78–0.94)***	0.80 (0.63–1.02)*	1.18 (0.78–1.78)	0.86 (0.76–0.98)**

°Elective caesarean section versus vaginal trial of labour, reference category.

In this analysis parity was regrouped into three categories instead of four, due to low numbers.

Stratification for three geographical areas:

^**a**^C4 = Urban areas;

^**b**^C6 = Semi-urban areas;

^**c**^Cx = Rural areas. Levels of significance:

* = p<0.10;

** = p<0.05;

*** = p<0.01.

Lastly, Table 5 shows the findings for birth setting in deliveries starting under supervision of the community midwife. Non-Western ethnicity was associated with high odds of delivering in an out-patient setting rather than at home in all geographical areas, mostly so in the Cx. Interestingly, the same pattern was present for women aged under 25, with a 40% excess in odds of delivering in an out-patient clinic. Overall, the association for SES with the odds of delivering in an out-patient clinic was U-shaped, with women from the middle SES quintile neighbourhoods being less likely to deliver in an out-patient clinic than women from low and high SES neighbourhoods. While in the overall analysis hospital density was strongly associated with more births in out-patient clinics, this effect disappeared after stratification into geographical areas.

**Table 5:** Multilevel logistic regression models of individual level maternal characteristics, neighbourhood SES and hospital density and location of birth° in low risk women. (Odds ratios, 95% confidence intervals in parentheses).

N = 1,363,343 births	Total	C4^a^	C6^b^	Cx^c^
Individual level	OR (95%CI)	OR (95%CI)	OR (95%CI)	OR (95%CI)
Maternal age (Ref. = 30–34 yr)				
Overall	***	*		***
≤ 24yr	1.44 (1.22–1.69)	1.47 (1.07–2.03)	1.61 (1.02–2.54)	1.44 (1.17–1.78)
25-29yr	0.99 (0.94–1.04)	1.06 (0.95–1.18)	1.08 (0.87–1.35)	0.98 (0.92–1.04)
>34yr	1.16 (1.13–1.20)	1.00 (0.94–1.08)	0.96 (0.83–1.11)	1.23 (1.18–1.27)
Parity (Ref. = multiparous 1 prior birth)				
Overall	***	***	*	***
Nulliparous	1.13 (1.11–1.15)	1.21 (1.17–1.27)	1.04 (0.97–1.11)	1.12 (1.10–1.14)
Multiparous (2–3)	0.72 (0.70–0.74)	0.77 (0.73–0.82)	0.90 (0.80–1.00)	0.70 (0.68–0.72)
Multiparous (≥3)	0.78 (0.66–0.93)	0.95 (0.68–1.32)	1.13 (0.69–1.85)	0.75 (0.60–0.93)
Ethnicity (Ref. = Western)				
Non-Western	4.26 (4.18–4.35)***	3.28 (3.14–3.42)***	3.89 (3.61–4.18)***	4.65 (4.54–4.77)
Neighbourhood level				
Socio-economic status (Ref.> 80th percentile)				
Overall	***		*	***
0-20th percentile	1.40 (1.28–1.54)	1.10 (0.85–1.41)	0.96 (0.78–1.19)	1.44 (1.29–1.60)
>20-40th percentile	0.99 (0.91–1.08)	0.92 (0.66–1.28)	0.91 (0.73–1.13)	1.00 (0.91–1.10)
>40-60th percentile	0.93 (0.85–1.01)	0.83 (0.56–1.23)	0.72 (0.53–0.99)	0.94 (0.86–1.03)
>60-80th percentile	0.82 (0.75–0.89)	1.14 (0.80–1.62)	0.76 (0.57–0.97)	0.82 (0.75–0.90)
Density	1.25 (1.11–1.40)***	0.89 (0.60–1.33)	0.77 (0.51–1.18)	1.03 (0.89–1.20)

°Location of birth: in an out-patient clinic (located in a hospital) versus elsewhere (at home or in a birthing centre, reference category).

Stratification for three geographical areas:

^**a**^C4 = Urban areas;

^**b**^C6 = Semi-urban areas;

^**c**^Cx = Rural areas. Levels of significance:

* = p<0.10;

** = p<0.05;

*** = p<0.01.

## Discussion

### Main findings of this study

Our multilevel analysis investigated utilization of obstetric care in a nine-year national dataset relating supply and demand. Results reject hypotheses that more supply (hospital density) induces more interventions in obstetric care in the Netherlands. SES and ethnicity effects also partially contradict common belief: adjusted for maternal factors and hospital density, living in low SES neighbourhoods and being from non-Western ethnic descent were not universally associated with higher odds of medical interventions. This finding was consistent across the interventions studied, with two exceptions: women from low SES areas in the rural areas were more likely to give birth in an outpatient clinic than their counterparts from more affluent areas, and referrals were more often in rural areas. If we accept the existing evidence on the increased risk for adverse birth outcomes for non-Western women and women living in low SES neighbourhoods, our results suggest relative underservice to the deprived in particular in larger cities, with a potential adverse impact on perinatal outcomes.

### What is already known on this topic

Higher supply is often associated with a higher probability of health care interventions and admissions [20]. Other studies contradict this [21]. In the Netherlands, Ravelli *et al*. have shown that for perinatal outcomes travelling time to facilities matters [22], suggesting that density might matter for outcome.

Inequalities in outcome may also be caused by inequalities in care utilization. Our findings on inequalities in care utilization rest on a large body of evidence [23–25]. Tudor Hart introduced the concept of the ‘Inverse Care Law’, meaning that those with low income -who need care most- receive least [26]. Studies within obstetrics that investigated the association between SES and care, have focused on time of entry into prenatal care and the uptake of prenatal screening rather than on interventions [27, 28]. Inequalities in utilization of obstetric care according to ethnic descent have also been described by many authors [27, 29, 30]. To our knowledge, no prior study included hospital density (as a proxy for supply), neighbourhood SES and ethnic descent (as a proxy for demand) in one analysis.

### What this study adds

Our analyses, stratified according to three geographical areas, show that hospital density is not associated with more health care interventions. The lack of an empirical effect of hospital density in this study may be caused by 1)a relative shortage of obstetric facilities, implying that all facilities are in full use; 2)the effect of guideline-led care, translating into uniform supply at the regional level; 3)factors affecting interventions somehow also affecting hospital density and 4)the current Dutch reimbursement system for obstetric interventions, which provides few economic incentives for ‘over-supply’ of care because it provides reimbursement for a whole ‘care process’ instead of a fee-for-service.

In absence of hospital density effects on the intervention rate, it was surprising to find inequalities according to neighbourhood SES and ethnicity. For such inequalities, several mechanisms have been postulated. First, the patients’ cultural background may influence preferences. There is a stronger tendency amongst non-Western women to prefer hospital based care and non-Western women may feel less aversion against medical interventions. While birth setting in the C4 confirms this tendency, this appears to be an exception. Hence, cultural background plays no role in the inequalities we observed. Secondly, patients’ unfamiliarity with the Dutch care system may underlie differences in intervention rates. Dutch patients are assertive in voicing their wishes to their physician and the same is probably expected from non-Western women [31]. If care providers are unaware of this difference in attitude, inequalities may arise. Lower levels of health literacy, which entails more than insufficient language proficiency, may add to this [32].

The inequalities in induction of labour and primary caesarean sections however suggest a role for care providers too. Care providers may not be conducive to specific high risk populations. Sparse consultation time can also make care providers less eager to thoroughly explain all treatment options. This may be enforced by health literacy issues of the patient, as mentioned above. Based on prior experiences, care providers may then make false assumptions on a patient’s risks or needs. In line with this, concordance in physician and patient ethnicity is associated with better perceived quality of care [33].

In our analysis the urbanisation level apparently acted as a confounder, with three distinct categories. We observed a striking difference between the C4 and the other areas: despite the increased prevalence of high risk groups and ample supply, the C4 surprisingly showed low intervention rates. We hypothesize that this could be one of the underlying mechanisms for the poorer perinatal outcomes in the large urban areas compared to the rest of the country. More research is needed to specify the influence of an unhealthy environment and social interactions within neighbourhoods.

A major strength of this study is the use of the complete 9-year national perinatal dataset with very high coverage that enabled us to map out demand and supply within obstetric care in the Netherlands. A previous study has shown that there is no need to correct for the record year because the outcomes are relatively stable across the years [34]. A second strength is that unlike most evidence on inequality in care utilization, we corrected for two important supply factors which may interact with the presence of deprived neighbourhoods and high migrant prevalence: hospital density and degree of urbanisation. The use of multilevel regression techniques enabled us to account for clustering of socio-demographic characteristics and other unknown effects (possibly health behaviours of women within neighbourhoods).

### Limitations of this study

Our study has several limitations. First, we had little individual level data on lifestyle or morbidity of the women. Therefore we were unable to take the women’s individual risk status into account and/or to adjust for it. Other factors that influence interventions such as patient preference, caregiver preference, or even other less tangible factors such as hospital policy could not be taken into account either. Hospital density was used as a proxy for density of all obstetric care providers. The Dutch system consists of both obstetricians and autonomously working community midwives. However, we do not have data on midwifery practice density at our disposal. Community midwives partly influence demand for hospital care by determining when to refer pregnant women under their supervision to hospital care. Referral is guided by the List of Obstetric Indications, which describes indications for referral [35]. Examples of these indications are haemophilia, hypertensive disorders, illicit substance abuse, and multiple pregnancies.

Also, the crude dichotomization of ethnic descent into ‘Western’ and ‘non-Western’ pools together diverse groups of women with different predispositions for adverse outcomes and possibly for interventions. Moreover, time spent in the ‘host’ country is of influence on language barriers and health literacy. Therefore data on migrant generation would have been desirable [36, 37].

Additionally, because ethnicity could be based on self-declared ethnicity, the registered ethnicity of two women with precisely the same mixed background, may differ because one may feel predominantly ‘Dutch’ whilst another might feel ‘Turkish’. This discrepancy may have influenced the effects we found in our analyses. However, this is likely to have led to a dilution and thus an underestimation of the true effect.

Finally, we assumed that women visit the hospital that is closest to their home. This does not always reflect true patient behaviour [38]. Attractive or repelling features of hospitals that we could not take into account may influence choice.

Despite the above mentioned limitations and the intrinsic limitations of observational data, the findings in this study give insight into the presence and size of inequalities in obstetric care utilization in the Netherlands. Further investigation is warranted to elucidate the underlying mechanisms, enabling the development of policy to reduce these inequalities.
